# Difficulties in Carotid Artery Stenting Due to Calcified Nodules: A Case Report

**DOI:** 10.7759/cureus.46233

**Published:** 2023-09-29

**Authors:** Kokyo Sakurada, Kiyofumi Yamada, Kisaki Amemiya, Eriko Yamaguchi, Hiroharu Kataoka

**Affiliations:** 1 Department of Neurosurgery, National Cerebral and Cardiovascular Center, Osaka, JPN; 2 Department of Pathology, National Cerebral and Cardiovascular Center, Osaka, JPN; 3 Department of Cerebrovascular Medicine, National Cerebral and Cardiovascular Center, Osaka, JPN

**Keywords:** carotid endarterectomy, calcified nodule, carotid artery stenting, carotid stenosis, carotid artery

## Abstract

The feasibility of carotid artery stenting (CAS) for carotid stenosis with severely calcified plaque remains controversial. Understanding the features associated with CAS difficulty in lesions with severe calcification is crucial. Calcified nodules, one of the morphological patterns of calcified plaques, have not been assessed for their association with the feasibility of CAS, even though they are associated with failure of percutaneous coronary intervention (PCI) in coronary arteries.

We present a rare case of carotid stenosis with calcified nodules in whom CAS was unsuccessful and who was subsequently successfully treated by carotid endarterectomy (CEA). A 79-year-old man presented with a transient ischemic attack caused by severe stenosis of the right internal carotid artery and opted for CAS. During the procedure, multiple attempts at balloon angioplasty using a 3.5-mm balloon were made, but effective dilation could not be achieved, resulting in recoil. Subsequently, the patient underwent carotid endarterectomy (CEA), and the excised specimen revealed a calcified nodule, a large nodular calcified plaque protruding into the lumen. The patient was discharged with a modified Rankin Scale score of 0 at 19 days after the CEA.

The protrusion of this large calcified nodule into the lumen was deemed responsible for the inadequate stent dilation. Although rarely reported in carotid stenosis, calcified nodules might represent a challenging plaque type for CAS treatment.

## Introduction

Carotid artery stenting (CAS) is an established treatment approach for carotid artery stenosis. Heavily calcified plaques have been reported to be associated with stent fracture and stent deformation during and after CAS procedures in recent reports [1]. However, there are some reports that CAS is feasible in treatments for lesions with severe calcifications [2,3]. The knowledge of features that are associated with difficulty in CAS in lesions with severe calcification is important.

Calcified nodules are one of the morphologic patterns of calcified plaques. In coronary lesions, calcified nodules are associated with a higher risk of unsuccessful percutaneous coronary intervention (PCI) than the other morphologies [4]. However, in the context of carotid artery lesions, the risk of treatment failure associated with calcified nodules has not been evaluated.

We present a rare case of a patient with carotid stenosis with calcified nodules for whom CAS was unsuccessful and who was subsequently successfully treated by carotid endarterectomy (CEA). The pathological features of this specific plaque morphology and the underlying mechanisms contributing to the failure of CAS for this morphology are discussed.

## Case presentation

**Figure 1:**
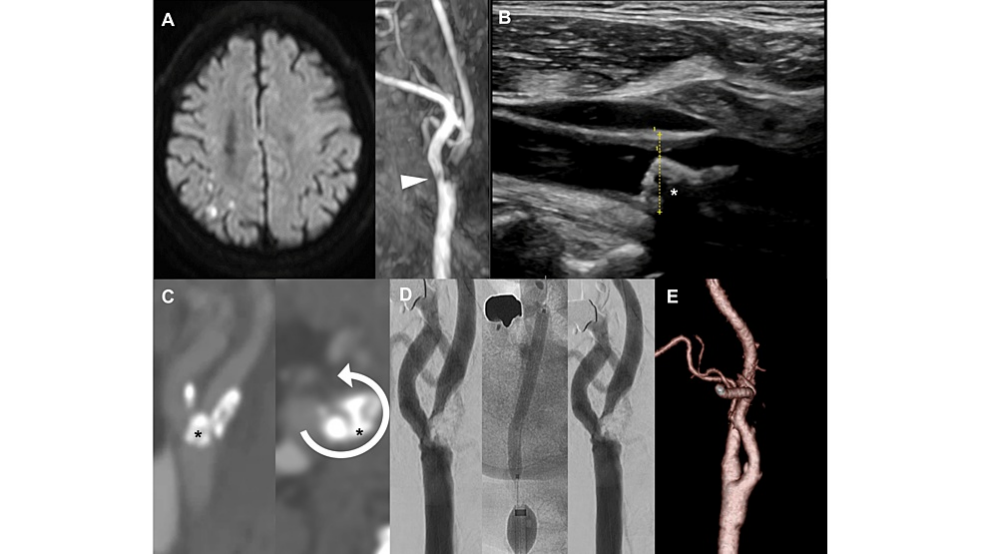
Neuroimaging and intraoperative findings (A) Cranial MR diffusion-weighted imaging performed one day after the presentation of transient dysarthria and hemiparalysis shows scattered high-intensity areas. The MR angiography shows a defect of flow signal in the ICA just distal to the bifurcation (arrowhead). (B) The ultrasound image shows a low echoic plaque protruding into the lumen of the ICA with an acoustic shadow (asterisk). (C) The CT angiography shows right carotid artery stenosis with an irregular luminal surface and a nodular calcification protruding into the vessel lumen (asterisk), and its circumference degree was 3/4 (arrow). (D) In the procedure of CAS, the stenotic lesion was immediately re-stenosed despite repeated balloon dilations. (E) The angiography one week after CEA shows a complete resolution of stenosis. ICA: internal carotid artery; MR: magnetic resonancy; CT: computed tomography; CAS: carotid artery stenting; CEA: carotid endarterectomy

A 79-year-old man was admitted to our institution due to transient dysarthria and weakness of the left lower extremity. He had a history of hypertension, hyperlipidemia, and angina. He had undergone percutaneous coronary intervention (PCI) 10 years previously. Scatter infarctions in the right hemisphere and severe stenosis of the right carotid artery were detected on magnetic resonance (MR) images (Figure 1A).

The stenotic lesion presented as a low echoic area with an acoustic shadow on ultrasonography, suggesting a calcified plaque (Figure 1B). Thin-slice computed tomography angiography (CTA) showed a calcified plaque with a nodule protruding into the lumen (Figure 1C).

We chose CAS for treatment for the following reasons: the right internal carotid artery (ICA) runs medially at 90 degrees to the external carotid artery (ECA); so-called twisted ICA was indicated to be difficult for CEA [5]; the calcification involving 3/4 of the vessel wall was indicated to be able to undergo CAS (Figure 1C) [2]; and the patient expressed a preference for CAS rather than CEA. We performed CAS after the administration of 75 mg of clopidogrel. Under local anesthesia, a 9-Fr balloon catheter (Optimo; Tokai Medical Products, Kasugai-City, Aichi, Japan) was deployed in the common carotid artery (CCA) on a right common femoral artery puncture. After a 4 mm Spider FX embolic protection device (Medtronic, Minneapolis, MN, USA) was opened at the cervical carotid artery for distal protection, a balloon (Rx-genity 3.5*40 mm; Kaneka Medical Products, Osaka, Japan) at the bifurcation was inflated to 8 atm for 30 seconds for pre-dilation. However, the lesion recoiled immediately after the deflation of the balloon. Although several additional dilations were attempted with larger balloons, sufficient dilation could not be achieved (Figure 1D). As a result, we decided to abandon the procedure.

**Figure 2:**
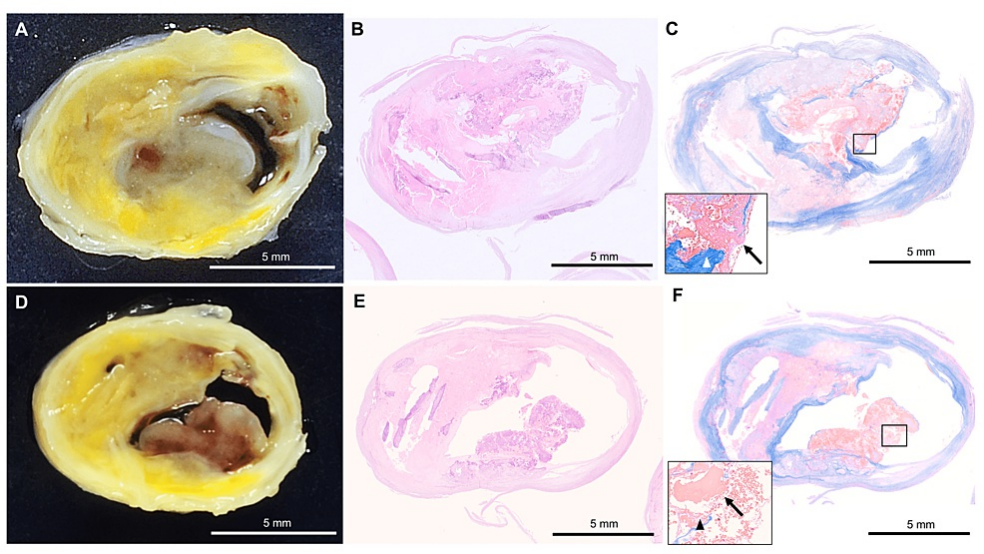
Pathological findings of the calcified nodule in the internal carotid artery (A, D) Macroscopically, the internal carotid artery serial cross-sections show an eruptive mass protruding into the lumen with an irregular surface. (B, E) The histopathological finding shows a calcified lesion with nodular calcification protruding into the lumen area on the hematoxylin-eosin (HE) stain. (C, F) The inset shows fibrous cap disruption (triangle) from eruptive calcific nodules associated with a fibrin thrombus (arrow) on Masson's trichrome staining. (B) and (C) are histological images corresponding to (A). (E) and (F) are histological images corresponding to (D). Scale bars: 5 mm.

Subsequently, we performed a carotid endarterectomy (CEA) and successfully resected a heavily calcified plaque. The plaque showed a large nodular calcified plaque protruding into the lumen (Figures 2A, 2D).

Pathological evaluation showed that the specimens were calcified nodules since the prominent nodular calcified plaques are not covered by fibrous caps in several locations (Figures 2B, 2C, 2E, 2F). Postoperative CT angiography showed complete resolution of the calcified plaque (Figure 1E). The patient was discharged with a modified Rankin Scale (mRS) of 0 at 19 days after the CEA.

## Discussion

We present a case of carotid artery stenosis with a calcified nodule that was refractory to CAS. Despite multiple attempts to dilate the lesion using a balloon, it remained resistant and immediately recoiled. The calcified nodule was confirmed through pathological examination following a subsequent carotid endarterectomy (CEA). The presence of a large calcified nodule protruding into the lumen was considered to be a contributing factor to the failure of balloon dilation.

Calcified nodules are defined as calcifications protruding into the lumen with accumulated fibrin [6]. It is one of the different types of calcified plaques: superficial, dense calcified plates; deep intimal calcifications; scattered microcalcification; and calcified nodules [7]. There have been few reports on the calcified nodules. Regarding the prevalence of calcified nodules in the carotid artery, according to imaging-based analysis, the presence was 7.9%-9.4% of carotid arteries [8,9]. There are only two case series providing pathological confirmation of calcified nodules in carotid arteries. Butcovan et al. and Diethrich et al. examined carotid endarterectomy specimens and reported the presence of calcified nodules in two out of 26 patients and five out of 14 patients, respectively [10,11]. Diethrich's report is the only study that evaluated the clinical course of calcified nodules, and it suggested an association between calcified nodules and previous neurological symptoms (66.7% vs. 33.3%, p < 0.05) [11]. There has been no study that has evaluated the association between CAS difficulties and calcified nodules in the carotid artery. In coronary lesions, calcified nodules are the least frequent cause of acute myocardial infarction (AMI) but are associated with a higher risk of unsuccessful PCIs than the other morphologies [4,12]. In our case of symptomatic carotid stenosis, CAS was performed at the first treatment, which was unsuccessful. Following a subsequent CEA, pathological examination confirmed the presence of a calcified nodule with fibrous cap disruption caused by eruptive calcific nodules, as well as the presence of a fibrin thrombus.

Despite being unsuccessful, we selected CAS as the initial treatment because severe calcifications are not necessarily associated with CAS failure. Tsutsumi et al. reported that CAS is feasible when the area of calcification occupies less than three-quarters of the circumference of the stenotic vessel [2]. They emphasized that CAS, or angioplasty, along with fragmentation of the calcified plaques, is necessary to achieve a sufficient luminal gain in heavily calcified circumferential lesions. Fragmentation of the calcified segment is achieved by the strong radial force exerted by stents and balloons [2]. However, in the present case, no cracks or fragmentations were seen in the resected calcified plaque specimen. Previous reports have indicated that PCI procedures for calcified nodules more frequently result in incomplete stent apposition compared to other plaque morphologies in coronary arteries. This is because a substantial amount of calcium protruding into the vessel lumen may hinder the conformability of a metal stent to the non-circular lumen geometry [12]. Based on our experience, the protrusion of the large calcified nodule into the lumen might make it impossible for the stent to conform to the non-circular lumen geometry and limit its ability to effectively exert radial force against the vessel wall.

In the present case, the non-contrasted thin slice CT imaging depicted the protruding plaque visibly. In previous reports, various imaging techniques were used to diagnose calcified nodules, including MR imaging, cone beam CT, and B-mode ultrasound [1,13]. In our case, the protruding calcification and the lumen were not detected on MR images because the blood flow signal was weakened due to severe stenosis.

Our case highlights the challenges associated with treating calcified nodules using CAS. The presence of calcified nodules may pose difficulties in achieving successful outcomes with this treatment approach. In the future, conducting further studies to evaluate the relationship between the presence of calcified nodules in the carotid artery and the failure of CAS would provide valuable insights.

## Conclusions

We present a case of carotid artery stenosis with calcification involving less than three-quarters of the vessel's circumference. Initially, we attempted treatment with CAS, but it was unsuccessful. Eventually, the patient was successfully treated with CEA. Histological examination confirmed the absence of a fibrous cap covering calcifications protruding into the vessel, and the presence of calcified nodules was confirmed. In this case, the presence of a large calcified nodule protruding into the lumen appeared to have contributed to the challenges encountered during the CAS procedure. This suggests that calcified nodules could be associated with difficulties in CAS procedures.
